# Supercritical CO_2_ Extraction of Oleoresin from Peruvian Ginger (*Zingiber officinale* Roscoe): Extraction Yield, Polyphenol Content, Antioxidant Capacity, Chemical Analysis and Storage Stability

**DOI:** 10.3390/molecules30051013

**Published:** 2025-02-22

**Authors:** Fiorella P. Cárdenas-Toro, Jennifer H. Meza-Coaquira, Monserrat Gonzalez-Gonzalez, Ceferino Carrera, Gerardo Fernández Barbero

**Affiliations:** 1 Chemical Engineering Program, Section of Industrial Engineering, Department of Engineering, Pontifical Catholic University of Perú, Av. Universitaria 1801, Lima 15088, Peru; jhmeza@pucp.edu.pe; 2 Department of Analytical Chemistry, Faculty of Sciences, Agrifood Campus of International Excellence (ceiA3), Wine and Agrifood Research Institute (IVAGRO), University of Cadiz, 11510 Puerto Real, Spain; d20020005@veracruz.tecnm.mx (M.G.-G.); ceferino.carrera@uca.es (C.C.); gerardo.fernandez@uca.es (G.F.B.)

**Keywords:** antioxidant capacity, extraction yield, ginger, gingerol, polyphenol content, shogaol, supercritical extraction, storage stability

## Abstract

In this study, we performed supercritical CO_2_ extraction of oleoresin from Peruvian ginger, focusing on the extraction yield, total polyphenol content, antioxidant capacity, and contents of gingerol and shogaol. The temperature (40 to 50 °C), pressure (80 to 250 bar), CO_2_ flow rate (2 and 8 ft^3^/h) and extraction time (10 to 360 min) were evaluated in three steps. The extraction yield was influenced by the temperature, pressure, flow rate and extraction time. Oleoresin extracts were obtained from 150 to 250 bar. The supercritical extraction conditions selected for the recovery of the oleoresin extract were 50 °C, 250 bar, 8 ft^3^/h and 360 min, resulting in an extraction yield of 25.99 ± 0.13 mg extracts/g dry basis, a total polyphenol content of 171.65 ± 2.12 mg of gallic acid equivalent (GAE)/g extract, an antioxidant capacity expressed as a half-maximal inhibitory concentration (IC_50_) of 1.02 ± 0.01 mg extract/mL methanol and a Ferric Reducing Antioxidant Power (FRAP) value of 368.14 ± 60.95 mg Trolox/g extract. The contents of gingerols and shogaols in the supercritical extract were 254.71 ± 33.79 mg of 6-gingerol/g extract, 24.46 ± 3.41 mg of 6-shogaol/g extract, 9.63 ± 2.51 mg of 8-gingerol/g extract, 51.01 ± 9.39 mg of 8-shogaol/g extract, 27.47 ± 5.06 mg of 10-gingerol/g extract and 20.11 ± 4.62 mg of 10-shogaol/g extract. There was no reduction in the total polyphenol content or antioxidant capacity according to the IC_50_ and FRAP assays, under storage conditions of 0 °C, 20 °C and 40 °C after 180 days; this indicates that the oleoresin obtained using supercritical CO_2_ extraction could be used as an additive in food products.

## 1. Introduction

Ginger (*Zingiber officinale* Roscoe) is a rhizome that is widely used as a seasoning in cuisine worldwide because of its pungent and spicy flavor due mainly to gingerols and shogaols, and its characteristic aroma related to volatile components (sesquiterpenes and monoterpenes). Ginger is also widely used in traditional Chinese medicine due to its pharmacological potential for cardiovascular protection, antioxidant activity, anti-inflammatory activity, antimicrobial activity and antiglycemic activity, as well as other protective effects [1,2,3]. Peru is one of the main exporters of ginger (*Zingiber officinale*) worldwide, with 54,000 tons in 2021 and significant growth in 2020 (217% in metric tons compared with 2016) during the coronavirus 2019 (COVID-19) pandemic because of its well-known immunological properties. Due to its ideal growing conditions, Junín is the main region in Perú for ginger cultivation, which benefits the Peruvian agricultural economy [4]. Considering the increase in ginger consumption in the local and international markets, it is possible to develop new value-added products from ginger that are of interest to consumers.

Ginger oleoresin is a dark brown viscous liquid with a characteristic odor and pungent flavor obtained from ginger rhizomes. It is composed of 22–31% essential oil, 0.5–8% total gingerols and 3–6% total shogaols. The main components of the essential oils are zingiberene, sesquiphellandrene, curcumene, *β*-phellandrene, d-limonene, *β*-bisabolene, α-farnesene, c-cadinene and germacrene, among others. 6-Gingerol is the most abundant gingerol compound in ginger oleoresin, followed by 8-gingerol and 10-gingerol. 6-Shogaol is a compound dehydrated from 6-gingerol after heating or storage and is found in ginger oleoresin, as are 8-shogaol and 10-shogaol [5].

Due to their low investment cost, steam distillation and solvent extraction are often used to obtain products from ginger. However, both techniques involve high temperatures during the separation of solvents, which can lead to the degradation of bioactive compounds, the high consumption of organic solvents, the presence of residual solvent in extracts, and the high consumption of energy due to the longer extraction time. Previous research has shown that environmentally friendly technologies, such as supercritical fluid technology, can be used to obtain products using a Generally Recognized as Safe (GRAS) solvent such as carbon dioxide; in this way, extraction yields and extraction qualities that are better or similar to those obtained using conventional techniques can be attained. Additionally, the supercritical fluid extraction technique is advantageous due to the low-temperature operating conditions that exist when carbon dioxide is used as a solvent (from 31 °C and 74 bar); this enables the thermosensitive components to be separated without degradation. In addition, the separation of the solvent via decompression allows an extract without residual solvent to be obtained. The appropriate selection and manipulation of the operating conditions during supercritical extraction allows extracts with different yields and qualities to be obtained [2,6].

Some studies have reported the supercritical extraction of products from ginger cultivated in countries such as Brazil, Indonesia and India. Mesomo et al. [7] studied the application of supercritical CO_2_ extraction to dried Brazilian ginger powder with a particle diameter of 0.63 mm. The operating conditions included temperatures of 20 °C, 40 °C and 60 °C, pressures of 100, 175 and 250 bar, a CO_2_ flow rate of 2 cm^3^/min, and an extraction time of 180 min. The results showed that temperature and pressure had a significant effect on the extraction yield, with the lowest extraction yield being obtained at 100 bar and 60 °C. The maximum extraction yield was 2.62% at 250 bar and 40 °C. The extract was characterized in terms of its content of α-zingiberene, *β*-sesquiphellandrene, α-farnesene, geranial, *β*-bisabolene and *β*-eudesmol. Compared with hydrodistillation extracts, the supercritical extracts exhibited greater inhibitory effects on Pseudomonas aeruginosa bacteria.

Said et al. [8] investigated the effects of various extraction methods on the extraction yield. Supercritical CO_2_ extraction was carried out at temperatures between 30 and 40 °C, pressures ranging from 150 to 250 bar and CO_2_ flow rates ranging from 10 to 20 g/min for 3 h. The highest extraction yield (6.87%) was obtained at 40 °C, 250 bar and a 15 g/min CO_2_ flow rate. They did not report the composition of the extracts.

Salea et al. [9] studied the application of supercritical CO_2_ extraction to dried Indonesian ginger powder with a particle diameter greater than 6 mm. They evaluated the effects of pressures ranging from 10 to 15 MPa, temperatures ranging from 35 to 45 °C, CO_2_ flow rates ranging from 10 to 20 g/min, a static extraction time of 1 h, and a dynamic extraction time of 4 h on the extraction yield and 6-gingerol content of the extract. The experiment revealed that, at constant pressure, the extraction yield decreased as the temperature increased. The highest extraction yield and 6-gingerol content were obtained at 15 MPa, 35 °C and 15 g/min, with a yield of 3.3% and a content of 22.3 mg/g extract.

Shukla et al. [10] studied the effects of a particle size ranging from 150 to 1204 μm, a temperature ranging from 35 to 50 °C, a pressure ranging from 150 to 300 bar, a CO_2_ flow rate ranging from 20 to 40 g/min, and a time ranging from 19 to 180 min on the extraction of products from dried Indian ginger powder. The highest extraction yield was obtained when the particle sizes ranged from 355 to 150 μm; a temperature of 40 °C resulted in the highest 6-shogaol content and an increased pressure resulted in the highest extraction yield. The optimal conditions for laboratory-scale extraction were 40 °C, 276 bar, a 30 g/min CO_2_ flow rate and a 153 min extraction time.

Studies related to the supercritical extraction of Peruvian ginger oleoresin and its characterization as well as the stability of supercritical oleoresin have not been reported. This work carried out a supercritical fluid extraction of oleoresin from Peruvian ginger. The effects of temperature, pressure, CO_2_ flow rate and extraction time on the extraction yield, total polyphenol content and antioxidant capacity according to the IC_50_ and FRAP assays were investigated in three steps. Ginger oleoresin extracted under the best conditions was characterized in terms of gingerols and shogaol contents, total polyphenol content and antioxidant capacity by IC_50_ and FRAP assays. A stability study was conducted under different storage conditions for 180 days.

## 2. Results

### 2.1. Raw Material Characterization

The moisture, ash, protein, and crude fiber contents of lyophilized Peruvian ginger were 4.6 ± 0.1%, 9.0 ± 0.0%, 6.4 ± 0.1% and 8.2 ± 0.2%, respectively. These values are slightly different from those presented in another study [11] in which the moisture, ash, protein and crude fiber contents were 9.0 ± 0.05%, 9.5 ± 0.12% and 7.2 ± 0.04%, respectively. The geometric mean diameter was 308.5 ± 1.7 μm.

### 2.2. Extraction Experiments

#### 2.2.1. Soxhlet Extraction

Soxhlet extraction using hexane as the solvent was performed at the boiling temperature of 69 °C over 6 h at a solvent/feed ratio of 10. This extraction time was considered adequate for exhaustive extraction of oleoresin from ginger using conventional solvents such as hexane as performed in other works [12]. The extraction yield X_0_ was 47.42 ± 0.97 mg extract/g dry basis. Similar results were obtained by [12] with extraction yield of 4.82 ± 0.23% at an extraction time of 6 h and solvent/feed ratio of 1/10, and [13], who reported an extraction yield of 4% at an extraction time of 9 h and solvent/feed ratio of 1/5. Furthermore, the total phenolic content, antioxidant capacity according to the IC_50_ and FRAP assays were 134.66 ± 4.96 mg GAE equivalent/g extract, 0.94 ± 0.06 mg extract/mL methanol and 426.90 ± 33.62, mg trolox/g extract, respectively. The chemical composition was 198.1 ± 21.9 mg of 6-gingerol/g extract, 5.6 ± 1.7 mg of 8-gingerol/g extract, 26.3 ± 4.0 mg of 10-gingerol/g extract, 21.0 ± 2.6 mg of 6-shogaol/g extract, 48.9 ± 7.4 mg of 8-shogaol/g extract and 36.1 ± 5.6 mg of 10-shogaol/g extract.

#### 2.2.2. Supercritical Extraction

##### Selection of Pressure and Temperature

Supercritical fluid extraction was conducted at temperatures of 40, 45 and 50 °C; pressures of 80, 100, 150 and 250 bar; and a CO_2_ flow rate of 2 ft^3^/h for 3 h of dynamic extraction. Table 1 shows the variation in the extraction yield under the studied conditions. The extraction yield varied between 1.90 ± 0.32 mg extract/g d.b. and 16.35 ± 1.10 mg extract/g d.b. under the studied conditions. The analysis of variance (ANOVA) revealed that the interaction between pressure and temperature had an effect on the extraction yield (*p* < 0.05). The Tukey test showed that the highest extraction yield was obtained at 50 °C and 250 bar (16.35 ± 1.10 mg extract/g d.b.), while the lowest extraction yield was obtained at 80 bar at all temperatures. It therefore appears that temperature and pressure affect the extraction solubility of ginger oleoresin due to the CO_2_ density and vapor pressure of the solute. According to Table 1, at constant pressure, the increase in temperature led to a reduction in density and solvation power; this effect was also observed for pressures of 80, 100 and 150 bar, with the oleoresin extraction yields being higher at 40 °C than at 60 °C. At pressures of 200 and 250 bar, the vapor pressure of the solute increases with the temperature, and this has a greater effect on the solubility of the ginger oleoresin; it was observed that the extraction yield increased more at 60 °C than at 40 °C.

**Figure 1:**
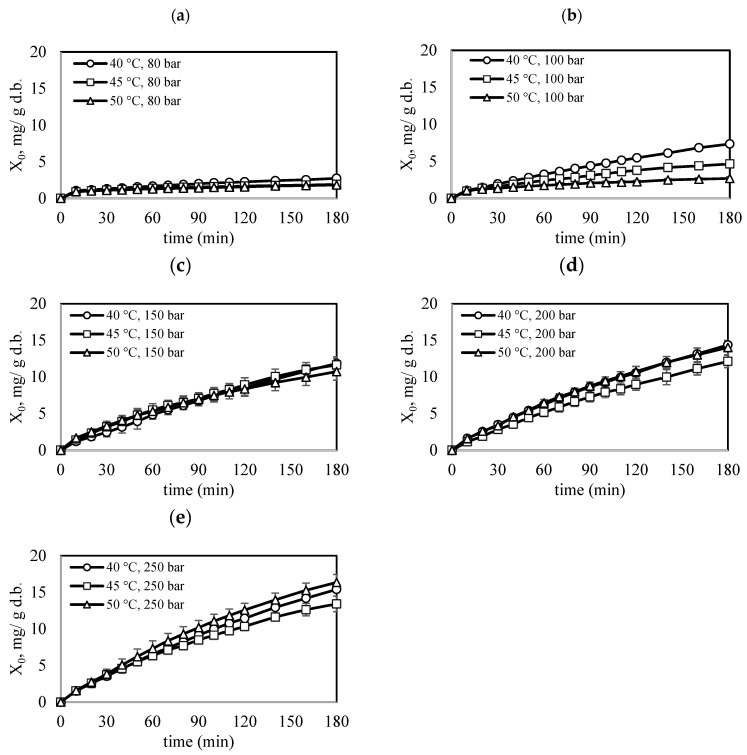
Overall extraction curves for the experimental conditions (**a**) P = 80 bar, (**b**) P = 100 bar, (**c**) P = 150 bar, (**d**) P = 200 bar, (**e**) P = 250 bar, CO_2_ flow rate of 2 ft^3^/h and total extraction time of 3 h.

Figure 1 shows the overall extraction curve (OEC) for the studied conditions. It consists of two different periods: a constant extraction rate (CER) period, where the mass transfer is governed by convection during the first 30 min, and a diffusion-controlled (DC) period, where the mass transfer is regulated by the diffusion mechanism. Crossover regions are also observed: at pressures up to 150 bar, the highest performance is observed at 40 °C, followed by 45 °C and 50 °C; at pressures of 200 and 250 bar, the highest performance is observed at temperatures of 50 °C, followed by 45 °C and 40 °C. The crossover phenomenon occurs because the vapor pressure of solute has a greater influence than the solvent density as the temperature rises at a constant pressure. Above the crossover pressure, the vapor pressure dominates, making the solute more soluble at higher temperatures. This phenomenon has been observed in other studies on supercritical extraction [9,10].

**Figure 2:**
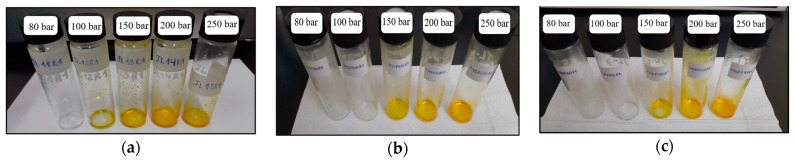
Color of supercritical extracts at studied conditions (**a**) 40 °C, (**b**) 45 °C and (**c**) 50 °C.

Figure 2 shows the color of the supercritical extracts under the studied conditions. The extracts obtained at 80 bar and 40 °C to 50 °C were translucent, as were those obtained at 100 bar and 45 °C to 50 °C, these extracts were considered essential oil extracts. The color of the extracts at 100 bar, 40 °C, and 150 to 250 bar was yellow-orange, and these extracts were considered as oleoresin extracts. These results are related to the high extraction yield of oleoresin, which is between 7.34 ± 0.18 and 16.35 ± 1.10 mg extracts/g dry basis at pressures ranging from 150 to 250 bar.

The bioactive properties of oleoresin extracts obtained at temperatures of 40, 45 and 50 °C, and pressures of 150, 200 and 250 bar were analyzed. Table 2 shows that the total polyphenol content varied between 1.48 ± 0.62 mg GAE equivalent/g extract and 215.07 ± 0.96 mg GAE equivalent/g extract, the IC_50_ varied between 0.85 ± 0.00 mg extract/mL methanol and 0.96 ± 0.07 mg extract/mL methanol, and the antioxidant capacity determined via the FRAP assay varied between 471.14 ± 3.03 mg trolox/g extract and 596.89 ± 38.22 mg Trolox/g extract. According to the Tukey test, the highest values for total polyphenol content and antioxidant capacity according to the IC_50_ and FRAP tests were obtained at 50 °C and 250 bar. From these results, the operating conditions of 50 °C and 250 bar for pressure and temperature were selected for the next step.

Supercritical extraction with carbon dioxide is commonly carried out at lower temperatures (40–60 °C) than Soxhlet extraction with hexane (69 °C). The use of lower temperatures preserves the temperature-sensitive bioactive components present in ginger roots, such as 6-gingerol. Furthermore, Soxhlet extraction requires the performance of subsequent solvent separation steps, such as rotary evaporation; this adds one more step to the processing and handling of the final extract. Meanwhile, the separation of CO_2_ in supercritical extraction occurs via phase change to a gaseous state, maintaining the purity of the extract. Finally, supercritical extraction selects bioactive compounds according to the temperature and pressure; this was evidenced by the analysis of the total polyphenol content and antioxidant capacity, as shown in Table 2.

##### Selection of the Flow Rate

The extraction yields at 50 °C, 250 bar and 180 min were of 17.40 ± 0.31 mg extract/g d.b. for a CO_2_ flow rate of 2 ft^3^/h and 19.18 ± 0.53 mg extract/g d.b. for CO_2_ flow rate of 8 ft^3^/h. One-way ANOVA and Tukey tests (*p* < 0.05) revealed that the CO_2_ flow rate affected the extraction yield, and the extraction yield for 8 ft^3^/h was greater than that for 2 ft^3^/h. The operating condition of 8 ft^3^/h for the CO_2_ flow rate was selected for the next step.

##### Selection of the Extraction Time

Table 3 presents the extraction yield and composition of gingerols and shogaols at 50 °C, 250 bar and 8 ft^3^/h over time ranging from 10 to 360 min (maximum operating time). According to the one-way ANOVA and Tukey test results (*p* < 0.05), the extraction time affected the extraction yield. In addition, the highest extraction yield was achieved at 360 min (25.99 ± 0.13 mg extract/g d.b.). Additionally, there was found no effect of extraction time on the contents of gingerols and shogaols in the extracts. From the presented results, an extraction time of 360 min was selected, which resulted in the highest extraction yield.

Table 4 shows a summary of the results obtained via supercritical CO_2_ extraction (50 °C, 250 bar, 8 ft^3^/h and 360 min) and Soxhlet extraction with hexane under the selected conditions; these results are presented in terms of the extraction yield, total polyphenol content and antioxidant capacity by IC_50_ and FRAP assays, as well as the content of gingerols and shogaols. Additionally, a comparison with other works is presented. The extraction yield obtained via supercritical extraction was lower than the value obtained via Soxhlet extraction; this is possibly because the exhaustive extraction time was not reached, as confirmed by the Tukey test when the effect of the extraction time was studied. The extraction yields were close to those obtained by the authors of [9,12], who performed extraction at 150 bar and temperatures of 60 °C and 35 °C, respectively. The difference in the extraction yields obtained from different sources is possibly due to differences in the initial content of oleoresin in the raw material, as well as its pretreatment prior to extraction. The quality of the oleoresin obtained via supercritical CO_2_ extraction and Soxhlet extraction was similar in terms of the total phenolic content and antioxidant activity; however, the contents of 6-, 8-, 10- gingerol and 6-, 8-shogaol were greater in oleoresin obtained via supercritical extraction than that obtained via Soxhlet extraction. This behavior was also reported in [9,10]. The use of extraction technology with supercritical CO_2_ can reduce the use of organic solvents such as hexane during Soxhlet extraction; lower energy consumption can also be achieved by working at a lower temperature and by recirculating the CO_2_ solvent, thus reducing the quantity of solvent consumed. In relation to the initial investment, supercritical CO_2_ extraction requires the use of specialized high-pressure equipment, which is more expensive than equipment that works at ambient pressure. However, the quality of the solvent-free product obtained via this extraction method could increase the industrial price of ginger oleoresin.

##### Stability Study

Table 5 shows the total phenolic content and antioxidant capacity, according to the IC_50_ and FRAP assays performed at storage temperatures of 0, 20 and 40 °C for 180 days, of oleoresin obtained at 50 °C, 250 bar, 6.74 g/min and 360 min. In all studied conditions, results showed that the best total polyphenol content and antioxidant capacity, according to IC_50_ and FRAP, were obtained at 45 and 90 days, according to the Tukey test. Storing ginger oleoresin at high temperatures can promote the conversion of gingerol to shogaol via the dehydration of a water molecule. Accelerated storage conditions, such as those occurring at 40 °C, can lead to the formation of 6-shogaol and the consequent formation of other components; for example, zingerone can be formed via the retro-aldol condensation reaction [14]. The storage of ginger oleoresin at 20 °C may reduce the formation of the 6-shogaol component, with this formation rate being lower at 0 °C. According to the results reported in the present study, as the storage time increases, the antioxidant capacity increases slightly, particularly when comparing storage times of 0 days and 180 days. This may be due to the formation of 6-shogaol from 6-gingerol, considering that 6-shogaol exhibits better antioxidant properties, according Dugasani et al. [15]. We can therefore conclude that the total polyphenol content and antioxidant capacity, according to the IC_50_ and FRAP assay, did not decrease over a time of 180 days at temperatures of 0, 20 and 40 °C.

## 3. Materials and Methods

### 3.1. Materials and Chemicals

Lyophilized ginger powder was obtained from La Joya Company in Arequipa, Peru. Carbon dioxide with 99.99% purity was supplied by Praxair Peru S.R.L. (Lima, Peru). Gallic acid monohydrate standard (≥98%), 6-gingerol standard (≥94%), and 6-shogaol standard (≥90%) were acquired from Sigma‒Aldrich (Saint Louis, MO, USA). All the solvents, reagents and standards used in analysis were of analytical grade.

### 3.2. Sample Preparation and Characterization

Lyophilized ginger powder was homogenized and sieved through a Tyler sieve 80 mesh (Series Tyler, W.S Tyler, Retsch, Haan, Germany). All powder less than 80 mesh was separated to prevent clogging during the supercritical extraction process. The samples were subsequently placed in plastic bags under vacuum and stored in freezer (Model FFHT2126LW4, Frigidaire, Mexico) protected from light until the experiments were performed. Lyophilized ginger powder was characterized in terms of moisture, ash, protein, and crude fiber content according to FAO (1986) in duplicate [16]. The geometric mean diameter (d_gw_) and the geometric standard deviation (s_gw_) were determined according to the American Society of Agricultural Engineers [17].

### 3.3. Extraction Experiments

#### 3.3.1. Soxhlet Extraction

A Soxhlet extraction with hexane as the solvent was performed to compare the performance with that of supercritical fluid extraction experiments. A total of 25 g of lyophilized ginger powder was placed into a 500 mL Soxhlet apparatus with 250 mL of hexane (solvent/feed ratio of 10). The extraction was carried out at the solvent boiling point of 69 °C for 6 h in triplicate. A rotary vacuum evaporator (Rotavapor R-3000, Buchi, Switzerland) was used to evaporate the solvent from the Soxhlet extracts.

#### 3.3.2. Supercritical Fluid Extraction (SFE)

This research was limited by the maximum operating conditions of the SFE equipment; this includes a pressure of 250 bar, a CO_2_ flow of 8 ft^3^/h and a time of 6 h.

##### Selection of Pressure and Temperature

The extraction experiment was performed based on the methodology described in a previous work [18]. A commercial SFE unit (SFT-150, Supercritical Fluid Technologies Inc., Newark, NJ, USA) was used in experiments. In total, 30 g of lyophilized ginger powder was placed into a 100 mL extraction vessel. The static extraction time was 10 min prior to the dynamic extraction step. For the dynamic extraction, the CO_2_ flow rate of 2 ft^3^/h for 3 h (solvent/feed ratio of 6.7). The extract was collected inside a glass flask immersed in an ice bath. The extract mass was measured with an analytical balance (LX 220 A, Precisa Gravimetrics AG, Dietikon, Switzerland) and collected in a flask. The final extract was stored in a freezer in the absence of light for further analyses.

Two-way ANOVA and Tukey tests (*p* < 0.05) were used to evaluate the influence of temperature and pressure on extraction yield, polyphenol content and antioxidant capacity by IC_50_ and FRAP assays. The experiments were carried out at temperatures of 40, 45 and 50 °C and pressures of 80, 100, 150, 200 and 250 bar in triplicate. The extraction temperature and pressure conditions that resulted in the highest extraction yield were selected for the next step. Table 1 shows that the CO_2_ density varies between 278 and 880 kg/m^3^. The density decreases with temperature at constant pressure and increases with pressure at constant temperature.

##### Selection of the Flow Rate

One-way ANOVA and Tukey tests (*p* < 0.05) were performed to determine the effect of flow rate on extraction yield. The supercritical extraction experiments were performed at CO_2_ flow rates of 2 ft^3^/h and 8 ft^3^/h (the equipment’s maximum capacity), extraction time of 180 min, and pressure and temperature described in Section 3.3.2: Selection of Pressure and Temperature in duplicate. The flow rate with highest extraction yield was selected for the next step.

##### Selection of the Extraction Time

One-way ANOVA and Tukey tests (*p* < 0.05) were performed to determine the effect of extraction time on extraction yield. A kinetic extraction experiment was performed from 10 to 360 min (maximum operating time) at the temperature, pressure and extraction time described in Section 3.3.2: Selection of the Flow Rate in triplicate. An analysis of total gingerols (6-, 8-, and 10- gingerols) and total shogaols (6-shogaol, 8-shogaol, and 10-shogaol) was performed. The extraction time with the highest extraction yield was selected.

Extraction experiments under selected operating conditions of pressure, temperature, flow rate and extraction time were performed to analyze the extraction yield, total polyphenol content, and antioxidant capacity by IC_50_ and FRAP assays and to study the extract stability under storage conditions.

##### Stability Study

Ginger oleoresin was stored at 0 °C, 20 °C and 40 °C to verify the variation in total polyphenol content, antioxidant capacity according to the IC_50_ and FRAP assay over a period of 0, 45, 90 and 180 days.

#### 3.3.3. Extract Characterization

##### Extraction Yield

The extraction yield (X_0_) was calculated according to Equation (1).

(1) $$X_{0\;}(\%)=\frac{m_{\mathrm{extract}}\;(g)}{m_{\mathrm{sample}}\;(g)\;d.b.}\times \;100$$

where m_extract_ is the mass of extract obtained at a given pressure and temperature, and m_sample_ is the mass of sample.

##### Total Polyphenol Content Analysis

The total polyphenol content of the extracts was determined according to the Folin‒Ciocalteu reagent method with slight modifications [19,20]. Approximately, 0.004 g of oleoresin was mixed with 20 mL of 70% (*v*/*v*) ethanol, vortexed at 1200 rpm for 1 min, and centrifuged at 3000 rpm for 10 min. A total of 0.5 mL of the supernatant was placed in a test tube, mixed with 2.5 mL of Folin–Cicolteau reagent solution (10% *v*/*v*) and vortexed at 1200 rpm for 5 s. Then, 2 mL of calcium carbonate solution (7.5% *w*/*v*) was added, and the mixture was vortexed at 1200 rpm for 5 s. The mixture was allowed to turn dark at 45 °C for 15 min in water bath followed by incubation at room temperature at 30 °C for 25 min. The solution was filtered with a 0.2 μm syringe nylon membrane filter (Acrodisc^®^, Pall Corporation, Show Low, AZ, USA). The absorbance was measured at 765 nm against a blank (ultrapure water). The calibration curve was constructed with gallic acid standard solutions in the range 2–200 μg GAE/mL (“μg GAE/mL = (Abs − 0.0877)/0.0102”, r^2^ = 0.9988). The total polyphenol content of the oleoresin extracts was expressed in milligrams of gallic acid equivalent per gram of ginger extract (mg GAE/g oleoresin). Analyses were performed in duplicate or triplicate.

##### Antioxidant Activity by 1,1-Diphenyl-2-Picrylhydrazyl (DPPH) Free-Radical Scavenging Assay

The DPPH free-radical scavenging activity of the extracts was determined according to the methods described by [21,22], with slight modifications. A 0.5 mM DPPH working solution with methanol was prepared daily and stored in the freezer until needed. The standard curve was prepared using gallic acid in the range of 0.0005 to 0.00275 mg/mL. Sample solutions were prepared by diluting 0.0099 g of extract in methanol, followed by successive dilutions to obtain concentrations of 0.05 to 0.495 mg oleoresin/mL methanol. An aliquot of 2 mL of sample mixture (extract or standard) was subsequently added to 0.4 mL of DPPH solution. The reaction mixture was stirred vigorously (1200 rpm × 5 s) and allowed to react for 30 min, after which the absorbance (A_1_) was measured at 517 nm with a UV-VIS spectrophotometer (G10S UV-VIS, Thermo Fisher Scientific, Shanghai, China). The initial absorbances of 2 mL of methanol (blank) and 0.4 mL of DPPH solution (A_0_) were measured at minute zero. Each sample was measured in duplicate.

The ability to scavenge DPPH radicals was expressed as the percentage of inhibition calculated via Equation (2):

% inhibition = (1 − A_1_/A_0_) × 100% (2)

where A_0_ is the absorbance of the blank at minute zero and A_1_ is the absorbance of the sample at 30 min.

A linear calibration curve was produced with r^2^ = 0.9881 (“% inhibition = 29,766 × gallic acid concentration (mg/mL) + 8.4809”).

IC_50_ was calculated from the regression equation obtained by plotting a graph of oleoresin concentration versus % inhibition.

##### Antioxidant Activity by Ferric Ion Reducing Antioxidant Power (FRAP) Assay

The total antioxidant activity of the extracts was determined via the FRAP assay, following the methodology reported by [23,24] with some modifications. The FRAP reagent was prepared with a mixture of 75 mL acetate buffer (300 mM, pH 3.6), 7.5 mL 10 mM TPTZ (2,4,6-tripyridyl-5-triazine) in 40 mM HCl, and 7.5 mL 20 mM FeCl_3_·6 H_2_O). A total of 0.0039–0.0044 g of extracts was diluted in 5 mL of absolute ethanol, and then an aliquot of 0.1 mL was diluted in 2 mL of absolute ethanol prior to analysis. A total of 0.2 mL of each diluted sample was allowed to react with 1.8 mL of FRAP solution (previously heated at 37 °C for 5 min) in a test tube for 40 min in a water bath at 37 °C in the dark. The solution was cooled at room temperature for 5 min. Each sample was filtered with a 0.2 μm syringe nylon membrane filter (Acrodisc^®^, Pall Corporation, New York, NY, USA). The absorbance was measured at 593 nm with a spectrophotometer (G10S UV-VIS, Thermo Fisher Scientific, Shanghai, China). Each sample was measured in duplicate. The calibration curve was constructed with a Trolox solution of 12–1500 μM as a standard (“μmol trolox/L = (Absorbance − 0.0879)/0.0044”, r^2^ = 0.996). The antioxidant activity was expressed as mg Trolox equivalent/g extract.

##### Analysis of Gingerols and Shogaols by Ultra-High Performance Liquid Chromatography Coupled to Diode Array Detector (UHPLC-DAD)

Oleoresin extracts obtained during the kinetic extraction experiment detailed in Section 3.3.2: Selection of the Extraction Time were analyzed according to the methodology described by [25]. For each sample, 0.015 g to 0.025 g of extract was diluted in 25 mL of methanol. The UHPLC system (Waters Corp., ACQUITYTM, UHPLCTM H-Class; Milford, MA, USA) was used for the analysis of the 6-gingerol, 6-shogaol, 8-gingerol, 8-shogaol, 10-gingerol and 10-shogaol. A UHPLC system was equipped with a quaternary pump, an automatic sampler, an oven column set at 65 °C for chromatographic separation and a DAD (Waters Corp., PDA100; Milford, MA, USA). The software application Empower3™ (Version 3.8.1) (Waters Corp.; Milford, MA, USA) was used for equipment control and data acquisition. A Waters ACQUITY UPLC BEH C18 (100 mm length, 2.1 mm internal diameter, 1.7 μm particle size) was used as the analytical column. The column was equipped with an ACQUITY Column In-Line Filter. The DAD detector was set to a 200–400 nm wavelength range for 3D scanning. For the peak integrations and quantification of the compounds, the DAD detector was set to 280 nm. Acidified water (0.1% acetic acid, solvent A) and acidified acetonitrile (0.1% acetic acid, solvent B) were used as the mobile phase, and a solvent flow rate of 0.5 mL min^−1^ and a column temperature of 65 °C were used. The gradient used for the chromatographic separations was as follows (time, % solvent B): 0 min, 0%; 0.1 min, 45%; 0.4 min, 45%; 0.9 min, 55%; 1.3 min, 75%; 1.8 min, 75%; 2.4 min, 100%; 4.4 min, 100%; and 4.6 min, 0%; followed by a 5 min column wash using 100% B. Extracts (0.015–0.025 g) extracts obtained from each sample were filtered through 0.2 μm nylon filters (Membrane Solutions; Dallas, TX, USA). Each sample injected had a 3 μL volume. The individual bioactive compounds were quantified based on the area of each of the chromatographic peaks corresponding to the gingerols and shogaols. The individual bioactive compounds identified and quantified in ginger included the following: 6-gingerol, 6- shogaol, 8-gingerol, 8-shogaol, 10-gingerol and 10-shogaol. A chromatogram (280 nm) of the chromatographic peak separation has been included in Supplementary Material (Figure S1). Compounds 6-gingerol and 6-shogaol were quantified based on the calibration curve corresponding to the standards at a concentration range of 0.1–240 mg/L for both compounds. The calibration curve for 6-gingerol was “Area = 4374.4 × Conc + 19073” (r^2^ = 0.9999; LOD: 0.18 mg/L; LOQ: 0.62 mg/L), whereas that for 6-shogaol was “Area = 7403.5 × Conc + 984.31” (r^2^ = 0.9995; LOD: 0.17 mg/L; LOQ: 0.57 mg/L). 8-Gingerol and 10-gingerol were quantified based on the 6-gingerol calibration curve, whereas 8-shogaol and 10-shogaol were quantified based on the 6-shogaol calibration curve, considering the molecular weight of each of the compounds. Based on the calibration curves generated for each one of the compounds of interest, the detection limits (LODs) and the quantification limits (LOQs) were determined by dividing 3 and 10 times, respectively, and the standard deviation of the blank sample by their respective regression slopes.

### 3.4. Statistical Analysis

The statistical analysis was performed using the software MINITAB^®^ v22.2.0 for analysis of variance and Tukey’s test to study the effect of the studied variables and determine significant differences at a significance level of 5% (α = 0.05).

## 4. Conclusions

In this research, the supercritical fluid extraction of Peruvian ginger was studied in terms of extraction yield, total polyphenol content, antioxidant capacity by IC_50_ and FRAP assay, chemical composition of gingerols and shogaols, and storage stability. Temperature, pressure, CO_2_ flow rate and extraction time had an effect on extraction yield under studied conditions. Oleoresin extracts were mainly obtained at 150, 200 and 250 bar. According to results, the best conditions for recovery of oleoresin extracts is 50 °C, 250 bar, 8 ft^3^/h and 360 min in terms of extraction yield is 22.88 ± 2.12 mg extracts/g dry basis. At this condition, polyphenol content of 171.65 ± 2.12 mg GAE equivalent/g extract, IC_50_ of 1.02 ± 0.01, FRAP antioxidant activity of 368.14 ± 60.95 mg Trolox/g extract. The composition of gingerols and shogaols were 254.71 ± 33.79 mg 6-gingerol/g extract, 24.46 ± 3.41 mg 6-shogaol/g extract, 9.63 ± 2.51 mg 8-gingerol/g extract, 51.01 ± 9.39 mg 8-shogaol/g extract, 27.47 ± 5.06 mg 10-gingerol/g extract and 20.11 ± 4.62 mg 10-shogaol/g extract. The storage of oleoresin extracts at 0 °C, 20 °C and 40 °C for 180 days showed that there was not a reduction in total polyphenol content or antioxidant capacity by IC_50_ and FRAP assay, showing that oleoresin extract obtained using supercritical CO_2_ extraction has a potential for application as additive in food products. According to the results of this study, due to the high antioxidant content of the ginger oleoresin obtained, it can be used in the food industry as a natural additive; this would improve the existing antioxidant properties of products valued on the market for their nutritional composition, such as olive oil enriched with ginger oleoresin and chocolate enriched with ginger oleoresin. On the other hand, the residue formed during supercritical extraction can be used in the baking industry to produce products that are based on wheat flour enriched with ginger flour.

## Figures and Tables

**Table 1:** Extraction yield for the studied pressure and temperature conditions.

T [°C]	P [bar]	Density [kg/m^3^]	Extraction Yield [mg Extract/g Dry Basis]
40	80	277.90	2.74 ± 0.13 ^g,h^
40	100	628.61	7.34 ± 0.18 ^f^
40	150	780.23	11.86 ± 0.91 ^d,e^
40	200	839.81	14.37 ± 0.39 ^a,b^
40	250	879.49	15.40 ± 0.24 ^a,b^
45	80	241.05	1.90 ± 0.32 ^h^
45	100	498.25	4.65 ± 0.38 ^g^
45	150	741.97	11.64 ± 0.91 ^d,e^
45	200	812.69	12.13 ± 0.85 ^c,d,e^
45	250	857.14	13.42 ± 1.06 ^b,c,d^
50	80	219.18	1.84 ± 0.07 ^h^
50	100	384.33	2.69 ± 0.23 ^g,h^
50	150	699.75	10.73 ± 1.17 ^e^
50	200	784.29	14.02 ± 0.99 ^b,c^
50	250	834.19	16.35 ± 1.10 ^a^

Different letters indicate statistically significant differences between the means of pairs compared (Tukey’s test) at a significance level of 0.05.

**Table 2:** Total polyphenol content and antioxidant activity by IC_50_ and FRAP assay for studied conditions of temperature and pressure.

T [°C]	P [bar]	Total Phenolic Content [mg GAE Equivalent /g Extract]	IC_50_ [mg Extract /mL Methanol]	FRAP Antioxidant Activity [mg Trolox /mL Methanol]
40	80	1.48 ± 0.62 ^f^	n.a.	n.a.
40	100	132.66 ± 18.69 ^d^	n.a.	n.a.
40	150	179.69 ± 11.99 ^b,c^	0.85 ± 0.00 ^c^	519.86 ± 59.41 ^a,b^
40	200	193.80 ± 19.17 ^a,b,c^	0.92 ± 0.00 ^a,b,c^	571.81 ± 15.91 ^a,b^
40	250	209.48 ± 21.34 ^a,b^	0.90 ± 0.02 ^a,b,c^	587.80 ± 79.95 ^a^
45	80	2.94 ± 0.00 ^f^	n.a.	n.a.
45	100	47.96 ± 15.33 ^e^	n.a.	n.a.
45	150	169.44 ± 2.83 ^c^	0.90 ± 0.02 ^a,b,c^	495.95 ± 16.36 ^a,b^
45	200	202.88 ± 0.38 ^a,b,c^	0.95 ± 0.00 ^a,b^	557.26 ±28.09 ^a,b^
45	250	215.07 ± 0.96 ^a^	0.92 ± 0.01 ^a,b,c^	594.40 ± 1.47 ^a^
50	80	4.24 ± 0.59 ^f^	n.a.	n.a.
50	100	1.99 ± 0.72 ^f^	n.a.	n.a.
50	150	179.89 ± 21.59 ^b,c^	0.86 ± 0.04 ^b,c^	471.14 ± 3.03 ^a,b^
50	200	197.74 ± 4.01 ^a,b,c^	0.87 ± 0.05 ^a,b,c^	539.12 ± 14.95 ^a,b^
50	250	204.57 ± 16.21 ^a,b^	0.96 ± 0.07 ^a^	596.89 ± 38.22 ^a^

n.a. not analyzed. Different letters represent statistically significant differences between the means of compared pairs (Tukey’s test) at a 0.05 significance level.

**Table 3:** Extraction yield and composition of gingerols and shogaols at 50 °C, 250 bar and 8ft^3^/h at different extraction times.

Time [min]	Extraction Yield [mg Extract/g d.b.]	Content of Gingerols and Shogaols in Oleoresin Extracts					
		[mg 6-Gingerol/g Extract]	[mg 6-Shogaol/g Extract]	[mg 8-Gingerol/g Extract]	[mg 8-Shogaol/g Extract]	[mg 10-Gingerol/g Extract]	[mg 10-Shogaol/g Extract]
10	3.08 ± 0.21 ^h^	269.5 ± 28.2 ^a^	25.2 ± 2.6 ^a^	8.3 ± 3.0 ^a^	49.5 ± 16.9 ^a^	26.6 ± 9.1 ^a^	16.0 ± 10.9 ^a^
30	6.64 ± 0.31 ^g^	250.5 ± 18.6 ^a^	23.6 ± 2.0 ^a^	7.7 ± 2.9 ^a^	46.2 ± 15.5 ^a^	24.9 ± 8.3 ^a^	19.0 ± 6.9 ^a^
60	10.37 ± 0.35 ^f^	246.3 ± 14.8 ^a^	23.3 ± 1.5 ^a^	8.1 ± 2.0 ^a^	47.3 ± 11.2 ^a^	25.4 ± 6.0 ^a^	20.4 ± 6.0 ^a^
120	15.47 ± 0.33 ^e^	244.3 ± 19.9 ^a^	23.3 ± 1.5 ^a^	8.4 ± 1.9 ^a^	49.7 ± 9.4 ^a^	26.8 ± 5.0 ^a^	22.6 ± 5.7 ^a^
180	19.09 ± 0.31 ^d^	244.2 ± 25.7 ^a^	23.2 ± 2.5 ^a^	8.7 ± 2.2 ^a^	51.4 ± 9.2 ^a^	27.7 ± 5.0 ^a^	23.1 ± 5.4 ^a^
240	22.07 ± 0.16 ^c^	246.3 ± 27.7 ^a^	23.5 ± 2.8 ^a^	9.1 ± 2.3 ^a^	52.3 ± 9.9 ^a^	28.2 ± 5.3 ^a^	22.6 ± 5.2 ^a^
300	24.15 ± 0.25 ^b^	249.1 ± 30.7 ^a^	23.9 ± 3.0 ^a^	9.4 ± 2.3 ^a^	51.6 ± 9.9 ^a^	27.8 ± 5.3 ^a^	21.3 ± 5.0 ^a^
360	25.99 ± 0.13 ^a^	254.7 ± 33.8 ^a^	24.5 ± 3.4 ^a^	9.6 ± 2.5 ^a^	51.0 ± 9.4 ^a^	27.5 ± 5.1 ^a^	20.1 ± 4.6 ^a^

Different letters denote statistically significant differences between the means of compared pairs (Tukey’s test) at a 0.05 significance level.

**Table 4:** Extraction yield, total polyphenol content, antioxidant capacity by IC_50_ and FRAP assays, and contents of gingerol and shogaol in extracts obtained at 50 °C, 250 bar, 8 ft^3^/h and 360 min for supercritical extraction and Soxhlet extraction.

	Supercritical CO_2_ Extraction (This Study)	Soxhlet Extraction with Hexane (This Study)	Supercritical CO_2_ Extraction [12]	Supercritical CO_2_ Extraction [9]	Supercritical CO_2_ Extraction [10]
Extraction conditions	50 °C 250 bar 6.74 g/min 360 min	69 °C 360 min	60 °C 150 bar 2 mL/min exhaustive extraction	35 °C 150 bar 15 g/min 240 min	40 °C 276 bar 30 g/min 153 min
Extraction yield	22.88 ± 2.12 mg extract/g d.b. ^a^	47.42 ± 0.97 mg extract/g d.b ^b^	1.54%	3.1%	8.6%
Total phenolic content	171.65 ± 2.12 mg GAE equivalent /g extract ^b^	134.66 ± 4.96 mg GAE equivalent /g extract ^b^	165 mg GAE equivalent/g extract	n.r.	n.r.
IC_50_	1.02 ± 0.01 mg extract /mL methanol ^b^	0.94 ± 0.06 mg extract /mL methanol ^b^	0.17 mg extract/mL ethanol	n.r.	n.r.
FRAP assay	368.14 ± 60.95 mg Trolox/g extract ^b^	426.90 ± 33.62 mg Trolox/g extract ^b^	n.r.	n.r.	n.r.
Composition (gingerols and shogaols)	254.71 ± 33.79 mg 6-gingerol/g extract ^b^ 9.63 ± 2.51 mg 8-gingerol/g extract ^b^ 27.47 ± 5.06 mg 10-gingerol/g extract ^b^ 24.46 ± 3.41 mg 6-shogaol/g extract ^b^ 51.01 ± 9.39 mg 8-shogaol/g extract ^b^ 20.11 ± 4.62 mg 10-shogaol/g extract ^b^	198.1 ± 21.9 mg 6-gingerol/g extract ^b^ 5.6 ± 1.7 mg 8-gingerol/g extract ^b^ 26.3 ± 4.0 mg 10-gingerol/g extract ^b^ 21.0 ± 2.6 mg 6-shogaol/g extract ^b^ 48.9 ± 7.4 mg 8-shogaol/g extract ^b^ 36.1 ± 5.6 mg 10-shogaol/g extract ^b^	22.30% 6-gingerol	20.69% 6-gingerol	25.98% 6-gingerol

n.r. not reported. ^a^ Results from 18 replicates. ^b^ Results from 3 replicates.

**Table 5:** Total phenolic content and antioxidant capacity by IC_50_ and FRAP assay of oleoresin at storage temperature of 0, 20 and 40 °C during 180 days for oleoresin obtained at 50 °C, 250 bar, 6.74 g/min and 360 min.

Temperature [°C]	Bioactive Properties	Storage Days			
		0	45	90	180
0	Total phenolic content mg GAE equivalent/g extract	169.3 ± 28.6 ^b^	230.4 ± 8.2 ^a^	187.4 ± 8.4 ^ab^	197.7 ± 10.4 ^ab^
	IC_50_ mg extract/mL methanol	1.0 ± 0.03 ^a^	0.96 ± 0.07 ^a^	1.07 ± 0.02 ^a^	1.2 ± 0.5 ^a^
	FRAP assay mg trolox/g extract	433.7 ± 85.0 ^b^	449.5 ± 13.6 ^b^	641.2 ± 13.9 ^a^	468.6 ± 82.8 ^b^
20	Total phenolic content mg GAE equivalent/g extract	162.4 ± 24.7 ^b^	210.6 ± 7.6 ^a^	189.9 ± 14.2 ^ab^	182.8 ± 13.9 ^ab^
	IC_50_ mg extract/mL methanol	0.90 ± 0.01 ^a^	0.90 ± 0.03 ^b^	0.90 ± 0.01 ^b^	0.94 ± 0.03 ^ab^
	FRAP assay mg trolox/g extract	357.6 ± 36.8 ^c^	441.6 ± 56.1 ^bc^	611.0 ± 40.1 ^a^	490.4 ± 6.5 ^b^
40	Total phenolic content mg GAE equivalent/g extract	183.2 ± 17.7 ^b^	250.4 ± 4.4 ^a^	187.3 ± 13.7 ^b^	193.2 ± 14.3 ^b^
	IC_50_ mg extract/mL methanol	1.03 ± 0.10 ^a^	0.96 ± 0.01 ^a^	0.93 ± 0.03 ^a^	0.97 ± 0.01 ^a^
	FRAP assay mg trolox/g extract	313.2 ± 44.5 ^c^	435.1 ± 8.6 ^b^	639.09 ± 19.8 ^a^	522.7 ± 73.9 ^b^

Distinct letters denote statistically significant differences between the means of compared pairs, as determined by Tukey’s test, at a 0.05 significance level for each bioactive properties and temperature across the evaluated times.

## Data Availability

The original contributions presented in this study are included in the article/Supplementary Material. Further inquiries can be directed to the corresponding author(s).

## Supplementary Materials

The following supporting information can be downloaded at: https://www.mdpi.com/article/10.3390/molecules30051013/s1, Figure S1: UHPLC-QToF-MS chromatogram of the six identified compounds. A: 6-gingerol; B: 6-shogaol; C: 8-gingerol; D: 8-shogaol; E: 10-gingerol; F: 10-shogaol.

## Abbreviations

The following abbreviations are used in this manuscript:

CO_2_	Carbon dioxide
GAE	Gallic acid equivalent
IC_50_	Half maximum inhibitory concentration
FRAP	Ferric reducing antioxidant power
GRAS	Generally Recognized as Safe
