# Supplementary material for: Supercritical CO2 Extraction of Oleoresin from Peruvian Ginger (Zingiber officinale Roscoe): Extraction Yield, Polyphenol Content, Antioxidant Capacity, Chemical Analysis and Storage Stability
Source: Molecules. 2025 Feb 22;30(5):1013. doi: 10.3390/molecules30051013 (PMC11901477; doi:10.3390/molecules30051013)
Supplement: Supplementary file 1 [file molecules-30-01013-s001.zip › molecules-3463113-supplementary.pdf]

Supercritical CO_2 Extraction of Oleoresin from Peruvian Ginger (*Zingiber officinale* Roscoe): Extraction Yield, Polyphenol Content, Antioxidant Capacity, Chemical Analysis and Storage Stability

Fiorella P. Cárdenas-Toro [1,*], Jennifer H. Meza-Coaquira [1], Monserrat Gonzalez-Gonzalez [2], Ceferino Carrera [2] and Gerardo Fernández Barbero [2]

Supplementary material

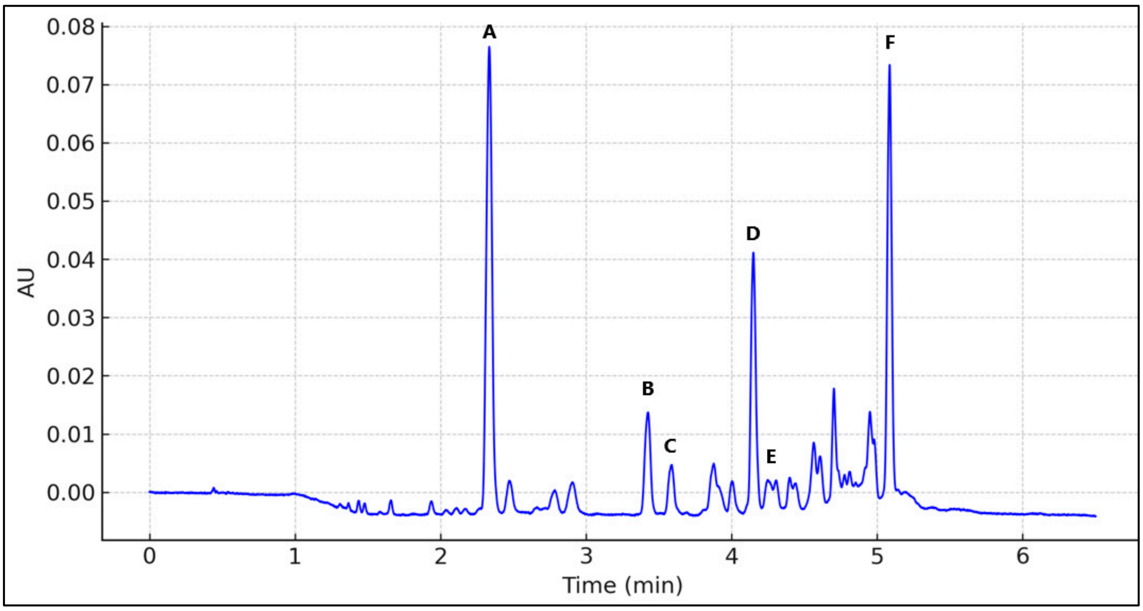

Figure S1. UHPLC-QToF-MS chromatogram of the six identified compounds. A: 6-gingerol; B: 6-shogaol; C: 8-gingerol; D: 8-shogaol; E: 10-gingerol; F: 10-shogaol.
